# Quality and women’s satisfaction with maternal referral practices in sub-Saharan African low and lower-middle income countries: a systematic review

**DOI:** 10.1186/s12884-020-03339-3

**Published:** 2020-11-11

**Authors:** Edward Kwabena Ameyaw, Carolyne Njue, Nguyen Toan Tran, Angela Dawson

**Affiliations:** The Australian Centre for Public and Population Health Research, Faculty of Health, University of Technology Sydney, Sydney, Australia

**Keywords:** Quality maternal referral, Maternal referral practices, Sub-Saharan Africa, Signal functions, Referral standards, women’s satisfaction, Referral guidelines

## Abstract

**Background:**

sub-Saharan African Low and Lower-Middle Income Countries (sSA LLMICs) have the highest burden of maternal and perinatal morbidity and mortality in the world. Timely and appropriate maternal referral to a suitable health facility is an indicator of effective health systems. In this systematic review we aimed to identify which referral practices are delivered according to accepted standards for pregnant women and newborns in sSA LLMICs by competent healthcare providers in line with the needs of pregnant women.

**Methods:**

Six electronic databases were systematically searched for primary data studies (2009–2018) in English reporting on maternal referral practices and their effectiveness. We conducted a content analysis guided by a framework for assessing the quality of maternal referral. Quality referral was defined as: timely identification of signal functions, established guidelines or standards, adequate documentation, staff accompaniment and prompt care by competent healthcare providers in the receiving facility.

**Results:**

Seventeen articles were included in the study. Most studies were quantitative (*n* = 11). Two studies reported that women were dissatisfied due to delays in referral processes that affected their health. Most articles (10) reported that women were not accompanied to higher levels of care, delays in referral processes, transport challenges and poor referral documentation. Some healthcare providers administered essential drugs such as misoprostol prior to referral.

**Conclusions:**

Efforts to improve maternal health in LLMICs should aim to enhance maternity care providers’ ability to identify conditions that demand referral. Low cost transport is needed to mitigate barriers of referral. To ensure quality maternal referral, district level health managers should be trained and equipped with the skills needed to monitor and evaluate referral documentation, including quality and efficiency of maternal referrals.

**Trial registration:**

Systematic review registration: PROSPERO registration CRD42018114261.

**Supplementary information:**

**Supplementary information** accompanies this paper at 10.1186/s12884-020-03339-3.

## Background

Timely referral to an appropriate health facility to address maternity needs is a key indicator of a functional health system [1, 2]. Efficient referral can result in a reduction of neonatal deaths with 18%, stillbirths with 27% and maternal deaths with 50% [3]. High quality referral is critical in low and lower-middle income countries (LLMICs) to prevent severe maternal morbidity that occurs in 8% of births in health facilities [4, 5]. In sub-Saharan Africa (sSA), obstructed labour, hypertensive disorders, unsafe abortion, sepsis and haemorrhage are the principal causes of maternal morbidity [6]. Multi-sectoral collaboration and well-coordinated levels of care, linking communities with essential maternal and newborn care is a necessary component of a high-quality referral system. Poor detection and treatment delays of complications is a major cause of maternal and perinatal mortality in LLMICs, particularly in sSA [7] where efforts are focused on improving standards of maternal and newborn referral [8, 9].

Maternity care that requires referral is usually due to complications that necessitate the use of life-saving services, or ‘*signal functions*’ as recommended by the World Health Organization (WHO) that cannot be provided by the referring facility [10]. Seven signal functions are recommended for basic emergency obstetric and neonatal care (BEmONC): administration of parenteral (1) antibiotics; (2) uterotonic drugs (e.g. oxytocin); (3) anticonvulsants (e.g. magnesium sulphate); (4) manual removal of retained placenta; (5) removal of other products of conception; (6) performing assisted vaginal birth and (7) neonatal resuscitation. Two additional services are recommended in the case of comprehensive EmONC (CEmONC) care: caesarean section and blood transfusion [10]. Any of these services may warrant referral.

According to WHO, all pregnant women should be correctly assessed upon presentation whether they can be attended to and if not immediately referred using a standard protocol. This should involve ‘appropriate information exchange and feedback to relevant health care staff’ [11]. Healthcare providers thus must communicate with the receiving facility in order for the woman to be managed efficiently. The receiving facility is also required to provide feedback on the outcome of the woman’s status [11].

Some international standards and guidelines are difficult to implement in some settings in sSA due to limited human resources, funding, cultural factors, being irrelevant, geographic challenges or unattainable in the context [11–14]. As a result, some countries such as Ethiopia, Ghana and Kenya have developed country-specific national level referral guidelines [15–17]. However, none of these is tailored towards maternal and newborn conditions.

Some systematic reviews on maternal and newborn referrals have been conducted in LLMICs, but these studies are based on evidence that is over a decade old [2, 18]. The review by Das, Gopalan excluded women who accessed the care [19]. Fikre [20] and Mahato et al. [21] limited their reviews to BEmONC, narrowing the scope of maternal and newborn care [20, 21]. None of these recent studies examined the quality of maternal and newborn referral practices and women’s satisfaction across sSA-LLMICs. Our research question was: “Which referral practices are delivered according to accepted standards for pregnant women and newborns in sSA-LLMICs by competent healthcare providers and in line with the need and wishes of women?”

## Methods

### Study design

This study was guided by the Population, Interventions, Comparators, Outcomes (PICO) framework [22]. Outcomes broadly fall under three key areas: quality of care (providers, referral system, information, medicines, and guidelines), satisfaction (women and family experience of referral) and effectiveness where available (health outcomes associated with referral). This systematic review was carried out following a priori protocol registered with PROSPERO [CRD42018114261].

A framework is a useful way to examine maternal and newborn referral, particularly in assessing elements of quality that are necessary for effective referral (S1). This framework has been adapted from the work of Hulton & Matthews and seeks to assess the quality of maternal referral by linking some key distinct components of institutional healthcare provision [23]. These components comprise health facility management, medicines and psychosocial support. Newborn referral is subsumed under maternal referral by the framework. The framework has two main components: 1. provision of maternal referral as provided by the health care system (supply) and 2. experience of referral as reported by women who utilise health facilities (demand). Most components under the supply section reflect elements of WHO’s health system building blocks indicating that strengthening maternal and child health care and referrals could also improve the health system in general [24].

The *Referral system* implies how referrals are initiated and conducted, from BEmONC to CEmONC or from one CEmOC facility to another CEmOC level of care. Referral practice should be in line with internationally accepted standards and relevant to contextual characteristics [10, 11]*. Human resources for health* as indicated in the framework refer to the availability, competency and motivation of healthcare providers to recognise and refer cases to the appropriate facilities in a timely manner. It also encompasses satisfaction of women about healthcare providers’ referral practices. *Maternity information systems* focus on the documentation of referred women or newborns including which data are collected, where and by whom. *Medicines and equipment* involve availability and appropriate use of essential lifesaving commodities for maternal and newborn health care such as the provision of a pre-referral loading dose of magnesium sulfate.

In terms of the demand side*, Cognition* focuses on whether women understood and were satisfied with the explanations given to them by healthcare providers. *Respect, dignity and equity* are concerned with whether women felt to have received respectful and equitable care. Women’s views about the level of cleanliness, availability of medicines and equipment in health facilities as well as value for money are mirrored by *satisfaction with facility, commodities and costs.* Lastly, *emotional support* is related with women’s perceptions of sensitive and responsive support by health care professionals.

### Data sources and search strategy

We searched six databases: African Journals Online (AJOL), CINAHL (Ebsco), Embase (Ovid), MEDLINE (Ovid), Pubmed and Scopus for quantitative, qualitative or mixed-method articles. The search focused on studies published in English between 2009 and 2018 and conducted in sSA-LLMICs according to the World Bank definition [25]. The following search terms were used: “maternal referral” OR “obstetric referral” OR “referral practice” OR “obstetrics” OR “pregnancy complications” OR “maternal health services” OR “maternal and child health” OR “emergency obstetrics and newborn care (EmONC)” OR “comprehensive emergency obstetric and newborn care (CEmONC)” OR “referral guidelines” OR “referral standards” OR “referral effectiveness” OR “signal functions” OR “women satisfaction” OR “family’s satisfaction” AND “sub-Saharan Africa” OR “Africa South of the Sahara” AND “low income countries” OR “lower-middle income countries”. Relevant references from bibliographies were hand-searched in order to identify all relevant peer-reviewed articles. All searches were conducted from 22nd October to 29th November 2018.

**Fig. 1:**
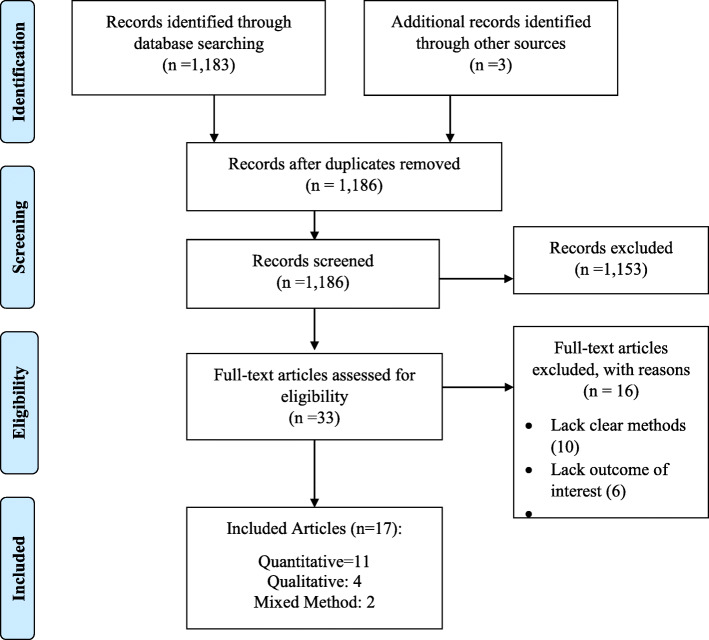
PRISMA 2009 Flow Diagram

Of the 1186 articles, 1153 were excluded after screening because of no outcomes of interest. A total of 33 were subjected to quality assessment after which 17 were included in our final analysis. Sixteen articles were excluded due to lack of clear methodology and limitations in the outcome of interest. The 17 articles included eleven quantitative, four qualitative and two mixed-method studies (Fig. 1) [26].

### Quality assessment and data extraction

The Critical Appraisal Skills Program (CASP) tool for qualitative research was used for assessing qualitative studies [27]. The McMaster Critical review form for quantitative studies was used [28].

### Data analysis

Content analysis [29] was applied guided by the framework for assessing quality of maternity care (Error! Reference source not found.) [23]. Content analysis involves systematic and objective identification of key characteristics of texts in order to make inferences [29].

The findings section of the papers were then scrutinised to identify key outcomes of interest, data extracted and examined related to the main components of the framework. These findings comprised direct quotations and quantitative data relevant to the main supply and demand components of the framework. Consequently, all quotations in the results section are from the original papers.

## Results

### Characteristics of included studies

Seventeen articles were included in this review. Study aim, context, methodology and relevant findings are summarised in Table 1. Most studies (*n* = 13) investigated the quality of referral from the healthcare providers’ perspective either through interviews or audit of health facility records [1, 8, 30–33, 35–37, 41–44]. Two studies explored women’s experiences of maternal and newborn referral [38, 39]. One study included both healthcare providers’ and women’s perspectives [40] whilst one South Sudanese study included perspectives of stakeholders from the local government sector, Faith-Based Organisations (FBOs), non-governmental organisations (NGOs), and community religious leaders in addition to healthcare providers [34]. Common conditions that prompted referral included premature rupture of membranes, obstructed labour and postpartum complications, such as haemorrhage and fistula [8, 34, 35, 37, 38, 40, 41]. Outcomes in these studies are summarised in S2.

**Table 1:** Summary of the 17 Articles

Reference	Country/Setting	Aim	Methods	Sample	Findings
Abodunrin et al., 2010 [30]	Nigeria (urban and rural communities in Ilorin, the capital of Kwara State)	To assess factors that determine referral practices of Traditional Birth Attendants (TBAs).	Descriptive quantitative survey: pre-tested semi-structured questionnaire	162 Registered TBAs, mean age = 46 years, 89.5% females, 71.6% married, 92% Islam, 64.2% had at least primary school education, 85.8% were part-time TBAs	▪ Identified timely and appropriate referrals among TBAs with more than one re-training (69.2%) and TBAs who have ever been visited by a supervisor (45%). Timely and appropriate referral was explained as referring women with high-risk pregnancies such as previous stillbirths, bleeding in previous or current pregnancies, multiple pregnancies, abnormal lie and not interfering with them. It also comprised immediate referral of women who had complications during labour management, such as bleeding during labour, prolonged labour, tiredness or loss of strength, seizures and retained placenta. ▪ Inappropriate referral comprised three conditions: delayed referral irrespective of the reason, wrong referral and non-referral. Delayed referral was defined as “not referring immediately any identified high-risk pregnancy and complicated labour.” ▪ Wrong referrals were those to any place other than a modern health facility. ▪ A significant relationship was found between initial source of skills acquisition, re-training, supervision and prompt/appropriate referral of high-risk pregnancies. ▪ Young and unmarried TBAs with higher education had a higher tendency of appropriate and timely referral. ▪ Most TBAs who started through inheritance usually refer late or not.
Afari et al., 2014 [31]	Ghana (4 health posts, 6 health centres and one district hospital in the Assin North Municipality)	To describe whether healthcare workers (HCW) identified systemic challenges and the significance of local engagement in developing strategies to enhance emergency maternity referral related processes.	Qualitative study: semi-structured interviews	18 HCWs (1 doctor, 2 emergency room nurses, 3 medical assistants, 4 community health officers, 8 midwives)	▪ Gaps in existing referral protocols-signal function recognition for referral, stabilising patients, initiating referrals, transportation arrangement for referral: “*Sometimes they hire commercial vehicles and sometimes too they use the motorbike. If there is no commercial vehicle at the station, they will beg someone to use their motorbike to convey them to the nearest health center or hospital, and then maybe somebody’s private car. The person might sacrifice*.” CHO, Health Post ▪ Few facilities adhered to national referral protocols. Poor referral documentation and lack of communication between sending and receiving facilities were reported, although national referral protocols existed: *“Apart from that [one] guy (HCW) who calls, the others don’t call so you’ll be here and such a case comes in. And […] with no…nobody accompanying… it’s really a challenge. Because if you know […] somebody is coming with eclampsia… you know you’re supposed to prepare first so that you receive [appropriately].”* Emergency Nurse, District Hospital *“Somebody who is fitting (or convulsing), a pregnant woman who is fitting… somebody (HCW) needs to accompany. But this is someone who is coming with relatives. They don’t know they have to turn the head to the side, [or] the person can aspirate saliva and any other thing[s]*.” Nurse, District Hospital ▪ HCWs recommendations: standardising implementation of the referral protocol, enhancing transportation, ensuring dependable data reporting and management systems, actively engagement of community and offering continuous training for health staff.
Akaba & Ekele, 2018 [32]	Nigeria (from either a primary, secondary, tertiary or private health facility to University of Abuja Teaching Hospital, Gwagwalada)	To determine maternal and fetal outcomes of emergency maternal referrals and reasons for these referrals.	Prospective longitudinal study-November 2015 to March 2016, data from case-notes, cross-checked with referral documentation when available.	All women requiring EmONC and referred from primary, secondary, tertiary or private health facility to University of Abuja Teaching Hospital, aged between 20 to 44.	▪ Nine cases (7.3%) were transported by ambulance. ▪ There was 8.9% emergency referral fatality rate (11 maternal deaths/123 maternal referrals). ▪ 7/11 maternal deaths occurred among women referred from secondary health facilities. ▪ Poor emergency maternity referrals and fetal outcomes were reported: 14 (11.5%) fresh stillbirths and six (4.9%) macerated stillbirths) due to late presentation.
Awoonor-Williams et al., 2015 [1]	Ghana (Upper East Region)	Maternity referral audit to strengthen the referral system for pregnant women and newborns in northern Ghana.	Quantitative, two-cycle prospective audit in early 2011and late 2011; questionnaire, 32 facilities in all-16 facilities, 12 health centres, 3 district and 1 regional hospital	223 referred women and their newborns (223 in each of the two cycles)	▪ Observed enhanced referral facilitative mechanisms-increased use of ambulances/vehicles for referrals (48 to 63%); higher usage of referral forms (66 to 77%); alerting receiving facilities through phone calls (38 to 65%); increment in feedback from receiving facilities (58 to 70%); all 6 women referred twice in the 2nd cycle were accompanied by health staff.
Carnahan et al., 2016 [33]	Tanzania (14 government facilities providing maternal health care, urban Dar es Salaam.	To investigate healthcare providers regarding prevention and management of postpartum haemorrhage (PPH).	Quantitative cross-sectional survey, questionnaire	102/115 (88.7%) nurses with midwifery training, 9 (7.8%) nurses without midwifery training, 4(3.5%) doctors/medical/ clinical officers from 10 dispensaries (60.9%), 2 hospitals (18.3%), 1 health centres (20.9%). 104/115 (90.4%) females, 71 (62.8%) with more than 6-year experience.	▪ All 14 facilities had referred 42.6% of women within the past three months. ▪ Forty-nine (42.6%) providers had referred at least one woman in the three months preceding the survey. ▪ 67.8% of 115 providers indicated consultation and referral communication systems are in place. ▪ 65.2% of 115 providers reported establishment of maternal referral transport system.
Elmusharaf et al., 2017 [34]	South Sudan (Renk County, Upper Nile State)	To ascertain patterns and contributory factors of pregnant women’s pathways from the onset of labour or complications until arriving in suitable health facilities.	Qualitative, Critical Incident Technique (CIT), Stakeholder Interviews.	28 key informants (2 from local government, 4 from county health department, 14 healthcare providers, 2 NGO employees, 3 Faith-Based Organisation (FBO) employees, 3 community religious leaders).	▪ Identified four referral pathways-late referral, zigzagging referral, multiple referrals and bypassing non-functioning facilities. ▪ Women who directly went to appropriate health facilities and by-passed non-functioning facilities survived. ▪ Competencies of healthcare providers and functionality of the initial point of care determined the pathway to further care. ▪ Trained midwives were found to be competent but TBAs were not.
Goodman et al., 2017 [35]	Ghana (Ridge Regional Hospital (RRH), Accra)	To describe maternal referrals received in Ridge Regional Hospital (RRH) and explore the timeliness with which women enter CEmOC.	10-week prospective cohort time-sequence information at arrival and from records and logbooks period from 9 to 9-2012 to 11-11-2012.	1082 women with pregnancy complications, 15–46 years, 0–8 parity, 24–49 weeks gestation age.	▪ Long waiting time upon arriving in receiving facility-40 min on average. ▪ The most distant referral facilities were 50 km from RRH. ▪ Gaps were identified in how maternal vital signs and labour assessments were recorded with 25 of 90 referrals found to be inappropriate. The most common reason for referral was for fetal-pelvic disproportion, however, fundal height was less than 40 cm, which does not support this diagnosis.
Kyei-Onanjiri et al., 2018 [36]	Ghana (120 health facilities across Upper East region)	To investigate the availability of emergency maternal care interventions in Upper East region.	Quantitative cross-sectional survey, questionnaire.	120 health facilities (9 public and private hospitals, 17 clinics, 41 health centres, 52 Community-based Health Planning and Service s (CHPS) centres, 1 maternity home).	▪ 94% health facilities were having standardised or printed referral forms for maternal referrals. ▪ 83% had a standard maternal referral procedure. ▪ 64% had shortwave radio/telephone for referral communication. ▪ 56% of facilities without shortwave radio/telephone could not access one within minutes in instances where it is needed. ▪ Most facilities always had a trained health provider. ▪ 73% had a midwife or doctor either on call or present at all times.
Mirkuzie et al., 2016 [37]	Ethiopia (10 public health centres with similar staff profile and providing EmONC, Addis Ababa)	To assess the proportion of maternal referrals resulting from premature rupture of membranes and investigate its correctness and management in Health centres.	Sequential explanatory mixed methods, routine retrospective data from birth and intrapartum referral logbooks and interviews, focused interview guide.	2820 women with maternal complications; 10 senior midwives	▪ All HC with high referral rates had many SBAs per caseload. ▪ 77.8% of the referred women had a spontaneous labour could have been wrongly referred because they were not in labour when they were referred. ▪ Some health centres observed women for about 8 h before referral initiation: “… *when we get mothers saying that their water has broken, after we evaluate them, they will be admitted to our health center and observed for about eight hours. If there is no spontaneous labour in eight hours we refer them to hospital after giving them a loading dose of Ampicillin… in the referral slip we write how long the mothers had been observed in our health center*.” (An informant from HC I).
Mselle & Kohi, 2016 [38]	Tanzania (Comprehensive Community Based Rehabilitation, a private, non-governmental organisation in Dar es Salaam)	To use women’s narratives to demonstrate the challenges leading to failure in accessing adequate maternal care in a timely manner	Qualitative, narrative research, semi-structured interview guide	16 women with obstetric fistula, aged between19 and 43 years, 82% rural dwellers, all unemployed, 88% had no or primary education	▪ Delay in making referral decision was reported: “… *it took 4 days at the village health facility, I could not give birth and then I was referred to the big hospital*” (Divorced, aged 29, Kibakwe, Dodoma). “…*In the health facility, I spend the night until morning … I had pains, the day passed, I slept again until morning again, and it was when a decision was made to transfer me to another hospital. They said it was because I had urine retention. On the third day is when I was transported to a big hospital*” (Divorced, aged 20, Mlandizi-Pwani).
Nuamah et al., 2016 [39]	Ghana (Antenatal clinics, Amansie West District in the Ashanti region)	To evaluate the role of socio-economic factors, perception and transport availability in fulfilling maternity referrals	Quantitative cross-sectional study, questionnaire	720 confirmed pregnant women from 5 sub-districts, 65.5% cohabitating, 28.8% married, 49.6% JHS/Middle School, 17% No formal education	▪ > 90% reported meeting staff in the receiving facility always. ▪ 76.6% disclosed that health staff in the receiving facility solved their problems. ▪ Most women were referred once and were not referred further. ▪ Commercial cars (88.2%) are more often used for referral than ambulances (6.6%).
Nwameme et al., 2014 [40]	Ghana (ante-natal care (ANC) clinics in Ga East district, Greater Accra region)	To examines the situation faced by women when they need emergency maternity care	Mixed Method, Questionnaire, In-depth Interview guide, Referral and facility review checklist	390 women attending ANC antenatal care clinic attendees and in-depth interviews with principal health care personnel, 17–46 aged women, 92% married, 44.6% Unorthodox Christians, 35.6% Orthodox Christians, 52.1% Junior High School, 12.8% no education, 76.2% traders, 43.6% parity 1, 29.7% parity 2	▪ Out of 17 women referred in their current pregnancies, none of them was sent by Ambulance, ten had public transport whilst seven made their own transport arrangement. ▪ Of the 17, fourteen got to the referral centre within 24 h, two within 48 h and one woman got there after 10 days. ▪ 15 had referral letters, but only 1 was accompanied by staff. ▪ Only one hospital had information computerized for easy access. ▪ Referring health facilities hardly received feedback: “*We don’t receive any feedback from the hospitals. At least it would help us understand what we could have done better.*” (In-depth Interview, Medical Officer) ▪ During maternity emergencies, they contact referral centres by mobile phone to find out if beds are available: “*There are hindrances between the two hospitals, no beds, no doctors…all these contribute to the delays*.” (In-depth Interview, Nursing Administrator).
Okafor et al., 2015 [8]	Nigeria (Semino Hospital and Maternity (SHM), Enugu State)	To audit childbirth emergency referrals by trained TBAs	Quantitative, retrospective, case records retrieved and data extracted with case record forms	205 women with childbirth emergencies, 41.5% rural dwellers, 58.5% urban dwellers, 90.2% married, 58.5% unemployed, 56.1% nullipara	▪ 155 (75.6%) of the women were delayed for more than 12 h before referral. ▪ 75.6% (155/205) arrived walking unsupported prior to admission whilst 24.4% (50/205) could not walk on admission.
Shimoda et al., 2015 [41]	Tanzania (urban, one regional referral hospital and one health centre in Dar es Salaam city)	To describe how midwives monitor and manage childbirth in order to achieve early consulting and timely referral to obstetricians	Qualitative, semi-structured interviews	11 midwives, 12.5 average year experience, 6 in the regional referral hospital, 5 in the health centre during the day, 4 with certificate, 4 with diploma, 2 with bachelor’s degree, 1 with master’s degree, 30–80 daily average births for their wards.	▪ Intrapartum management and monitoring/examination to arrive at referral decision consisted of 3 phases: 1) initial encountering, 2) monitoring, and 3) acting that finally resulted in referral. ▪ Prompt referral upon identifying signal function beyond the facility’s capacity: “*When she put in the catheter, we saw some blood starting to pass. That is the sign of obstructed labor. That’s why I decided to refer immediately*.” (F) ▪ In instances where mother and fetus conditions are worsening, midwives decide earlier without taking time to confirm labour.
Strand et al., 2009 [42]	Angola (3 peripheral birth units-Cazenga, Palanca and Sambizanga)	To assess the efficacy of the newly established network of peripheral birth units and their linkage to hospitals.	Two-phase quantitative survey, review of maternal records	157 referred women for 1st and 92 for 2nd phase, 24.1 mean age, 36% < 20 years, 43% primiparae, 32% ≥4 previous births in 1st phase	▪ 157 deaths (17.8% case fatality rate) occurred among traced referrals in the first phase, no maternal death in the second phase. ▪ Redacted proportion of referred women who were left without medical evaluation/treatment observed from the women’s records (45% in the first phase of the study to 27% in the second phase (*p* = 0.007).
Tayler-Smith et al., 2013 [43]	Burundi (rural district, Kabezi)	To describe Medecins sans Frontieres’ communication and ambulance service, examine relationship between referral time and adverse outcome, explore effect of referral service on coverage of complications and caesarean sections.	Cross-sectional study, retrospective analysis	1478 ambulance call-outs/referrals.	▪ Median referral time (time between call-out to the ambulance returning with the patient at CURGO) was 78 min. ▪ One maternal death occurred among referred women but it was not possible to evaluate the linkage between death and referral time. ▪ 3-h referral duration or higher was associated with increased risk of early neonatal mortality-15% as compared with 9%
Windsma et al., 2017 [44]	Ethiopia (20 health centres in the Eastern Gurage Zone)	To assess BEmONC, knowledge of high-risk pregnancies and referral capacity in health centres	Cross-sectional survey	37 healthcare providers (18 heads of health centres, 14 midwives, 3 nurses, 1 health officer, 1 other), 45 months average of experience among heads with 27.5 median age, median age of 24 years for others with 24 months average experience.	▪ Most staff used their own mobile phones for referral correspondence-only 5 facilities (26.3%) had a working landline and 1 (5.3%) facility had a mobile phone. ▪There were 5 ambulances for the Eastern Gurage Zone population: Two stationed in health centres and 3 in District Health Offices. ▪ Distance to the referral Butajira General Hospital used by all health centres was 16.5 km on average. ▪ There is a need to train staff of the health centres in the identification of signal functions and BEmONC.

### Provision of maternity referral

All 17 studies reported on various aspects of maternal and newborn referral and are presented under the sub-themes as outlined by the supply side of the framework for assessing the quality of maternal referral.

#### Referral system

Two studies indicated use of standard maternal and newborn referral procedures, availability of standardised referral forms and health provider escorts [1, 36]. Awoonor-Williams & Bailey [1] indicated that such procedures were accompanied by telephoning ahead to prompt the receiving hospital.

In that study in 16 facilities in Ghana, healthcare providers escorted all referred women and their newborns from health centres by ambulance to BEmOC and CEmOC facilities [1]. In two other studies in Ghana, health care providers rarely escorted women [31, 40]. Some referrals were unaccompanied because these were not emergencies [40]. Unaccompanied women sometimes arrived late [40].

Elmusharaf et al. [34] identified four referral pathways (Table 1) [34]. One woman narrated her zigzag referral:

*Family members lifted the pregnant woman onto a donkey-driven cart and went to the village’s medical assistant. When they arrived, her water broke. The medical assistant prescribed drugs and told them that she was in labour and that the midwife should deliver her straight away. He sent them back to the midwife for birth. After spending three hours with the midwife without progress, the pregnant woman was exhausted. The midwife advised them to go back to the medical assistant. They spent most of the night going back and forth between a midwife and a medical assistant until the midwife insisted on the medical assistant referring them to hospital* (5JMD) [34].

In a study from Angola delays of up to 13.7 h were noted in the triage of referred women upon arrival in the receiving facility [42]. Referred women and their newborns were simply added to the queue with other patients. When healthcare providers were alerted about this issue, a meeting was held to identify referred women and prioritise them upon arrival. After this strategy was implemented, they spent an average of 9 min to meet a midwife and 71 min to be assessed by a doctor.

In Ghana, triage initiation for women in labour was found to vary by shift or timing of the day in one hospital [35]. Average waiting time in the morning was 35 min, 28 min in the evening and 55 min in the night. For a woman in the first stage of labour, it took an average of 35 min for triage to take place and 24 min after this for her to be admitted to the labour ward. Those in the second stage were evaluated within 30 min and moved to the labour ward within 10 min [35].

In Burundi, a 15% higher chance of neonatal deaths was observed for women who took ≥3 h to arrive in the next facility. Facilities had to call for an ambulance from another facility which delayed in some instances [43]. In the absence of ambulances, donkey-driven carts, tractors*,* pick-up trucks, public transport (taxis and lorries), motorised tricycles and motorbikes were used [31, 32, 36, 39, 40, 44].

Some health facilities were not equipped with telephones and some healthcare providers from primary health centres in southern Ethiopia had to use their personal phones to notify receiving facilities about referrals [44]. A communication system for facilitating referrals in 14 government health facilities in Tanzania was reported without details [33].

Referring health facilities are not always given feedback so that opportunities for improvement did not reach these [31, 40]. High workload of health care workers was cited as the main reason why feedback was not given. A Medical Officer from Ghana reported:

“*We don’t receive any feedback from the hospitals. At least it would help us understand what we could have done better.*” (In-depth Interview, Medical Officer) [40].

However, in one study, feedback on referral was given verbally by the women or their families [1].

#### International standards for the management of maternity emergencies

Nwameme & Phillips [40] indicated that more than 75% of staff from two of three facilities were trained how to use national referral guidelines. Two of the three studies reporting availability of national referral guidelines in Ghana indicated adherence in health facilities [1, 40]. Adherence includes documentation of referral indication, telephoning the referral facility ahead of time and arranging transportation, preferably by ambulance. In a survey of 120 health facilities in Ghana, 94% of the facilities had standard referral forms and 83% followed a standard referral procedure [36].

In Tanzania, guidelines for midwifery care were used in the studied hospital and health centre to manage intrapartum monitoring leading to emergency referrals of 11 women with prolonged labour [41]. Unlike WHO guidelines and recommendations, the national guidelines of Ghana do not mention that uterotonics should be applied to reduce haemorrhage [11, 16].

#### Human resources for health

A variety of providers were involved in maternity referrals, but few details were available regarding their experience and training [1, 30, 31, 33, 36, 39–41, 44, 45]. Three papers reported that midwives and nurses had between two and 12.5 years of experience [41, 44] and doctors and nurses with midwifery training had at least 6 years [33].

In 14 government health facilities in Tanzania, 17% of 115 health care providers could correctly diagnose post-partum haemorrhage (blood loss ≥500 ml, or blood loss of 500 ml with shock symptoms) [33]. Almost all healthcare providers (98.3%) knew that misoprostol can be used for post-partum haemorrhage (PPH), but only 62.6% was able to state the recommended dose (600 μg) and 36.5% were able to prescribe it, because it was not always available [33]. No significant differences were found in the mean scores of PPH-related knowledge between providers who had > 6 years of experience or less.

The 268/350 (76.6%) women who were referred to a higher level facility in Ghana reported that healthcare providers were competent enough to solve their problems [39]. In Kwara State in Nigeria, 128 (79.0%) Traditional Birth Attendants (TBAs) indicated that they did not refer in a timely manner [30]. These were untrained TBAs who acquired their skills through self-initiation or inheritance. Nine of the twenty TBAs who had at least one supervisory visit by a qualified provider were able to conduct appropriate referral. In addition, nine out of the thirteen TBAs who had attended > 1 training course, referred women with complications appropriately [30].

Okafor, Arinze-Onyia [8] also reported that trained TBAs in Nigeria delayed referral for women with signs of difficulty in childbirth for > 12 h [8]. A senior manager in a Reproductive Health and Midwifery Department in South Sudan complained about their competencies:

*In the past, TBAs have arrived in Renk hospital with pregnant women with their babies partly delivered; parts of the foetus, such as the head, the arm or the leg, outside the woman’s body and the rest of the body still inside*. (Senior Manager) [34].

Poor skills of lower level doctors and midwives were reported in Ghana:

“*Last time a pregnant woman came here …*. *And I was saying but there is a doctor at your place, so why did you rush here without a midwife accompanying you, and she said ‘Auntie, I had been admitted there for a long time. And each time the doctor came, he said let’s wait a bit more, and I was experiencing a lot of discomfort, and insisted that they discharge me, so they finally reluctantly discharged me.’ And when she arrived here, true, it was twins. But one was IUFD (macerated) already. So she was able to get the first twin alive.*” (Midwife, District Hospital).

Health professionals noted the need for ongoing professional development. One midwife said:

*“They (staff in the district hospital) need refresher courses... They should allow them to go to workshops so that they will see what is going on …. Me, I always learn from my junior nurses and midwives because I joined it [midwifery] about 10 years ago, and things are changing. Even the instrument [s] we are using [are] changing.”* (Midwife, Health Centre) [31].

#### Maternity information systems

Some health facilities in Ghana routinely used logbooks, care plans, referral letters and forms or slips correctly as required by the national referral guidelines [16]. Poor referral documentation was reported in Ghana where only six out of 11 sampled health facilities had referral registers and details concerning indications and treatment, while current status and treatment in the receiving facility were lacking and only one EmONC facility had a computerised referral information system [31, 40].

#### Medicines and equipment

In the seventeen studies, four included information concerning medicines and equipment such as misoprostol [1, 35, 37, 46]. A decline in correct partograph use was reported in Ghana [1]. Audit in peripheral health facilities in Angola revealed poor quality of partographs without further detail [42]. In Tanzania, midwives correctly used catheters to enhance referral decision making*.*

### Experience of referral care

No article provided insights into cognition and emotional support during referral*.* In Tanzania, some women in a primary health facility indicated that doctors were not readily available to check progress and refer to EmOC facilities, if necessary [38]. One woman narrated her story:

*“When we got to the dispensary nurses told me to wait. At 8 pm labour pains became intense, I started pushing but the baby could not come out, and the doctor was not around. Next day I continued pushing the whole day again until at around 8 pm when the doctor came …*” (Divorced, aged 33, Mbori Dodoma), [38].

Just over 10% (*N* = 390) of women in three health facilities in Accra Ghana indicated that poor attitudes of nurses were a source constraint to referral [40]. In that study 180/390 women (46.2%) complained about costs while three reported previous bad experiences as sources of dissatisfaction and constraints to referrals without further details [40].

### Socio-cultural factors affecting women’s adherence to referral

While the framework did not include socio-cultural factors, these were identified as having an effect on women’s referral for care. In Ghana socio-cultural beliefs relating to fear of blood transfusion and fear of death in higher level facilities affected women’s desire to travel to next level facilities [44]. In a CEmOC health facility in Ghana, 720 (57%) women had to consult their husbands for permission to follow health professionals’ advice [39]. For some women in rural Tanzania with obstetric fistula, the decision to travel to the next facility was made by their uncles, grandmothers, husbands and mothers-in-law [38].

## Discussion

Mere existence of referral guidelines does not imply their full application in maternal and newborn referrals in sSA LLMICs. Application of guidelines is hindered by heavy workloads, low competence of health care providers and non-availability of ambulances [13, 31, 34]. This requires greater policy attention and reaffirms lack of competent staff and essential medicines for managing maternity complications [45–48]. Strategies such as task sharing may be useful and involve upscaling of lower cadres of staff to conduct assessments to refer promptly or immediately receive women for treatment [49].

Few quality referrals were reported among lower-level midwives and nurses in clinics, health centres and medical centres [40, 41]. Training and supervision were, however, associated with higher quality referrals [32]. For TBAs, our findings reinforce the position of Sibley &, Sipe that TBAs need to be trained to refer women [50]. Strong teamwork and collaboration between TBAs, lower and higher level health care providers could result in better outcomes [51]. Our findings corroborate the need to train district level managers in the skills needed to monitor the effectiveness of maternal and newborn referrals [52]. Consistent professional development is needed for all health workers to identify and manage complications and refer in a timely manner during all stages of labour [53].

Some women bypassed lower level facilities because they knew about potential delays in referral decision-making by lower level health care workers as noted in other studies [54, 55]. Other studies similarly showed that such delays as well as delays in triage initiation in referring facilities compromise the quality of referral [9, 56]. Regular monitoring and evaluation of referral processes especially at district level may be essential in improving referrals. Such activities can focus on specific indicators such as triage duration, referral decision-making competencies, referral outcomes and availability of means of transport for all maternity emergencies [52].

Using tools such as phone calls depending on feasibility and geographical context can be employed to facilitate referrals. Toll-free numbers for mobile phones and Geographic Information Systems (GIS) based transport have been used to coordinate and facilitate referral and transport arrangements [57–63].

Poor referral record keeping is consistent with other studies resulting from limited knowledge of health care providers or financial constraints [31, 64, 65]. Mechanisms that encourage health care providers to have detailed documentation, in addition to user-friendly forms and provider training on the relevance of documentation could improve this.

The instrumental role of free or low cost, safe and reliable transport, preferably with ambulances, has been widely acknowledged [66–69]. Motorcycle ambulances could be used as relatively cheaper options [70]. Our findings corroborate the need to strengthen transport for maternal and newborn referral free of charge to women and their families [71]. An efficient, low cost transport system provides women with positive referral experiences [72]. The cost of transportation to the referred facility may dissuade women from adhering to referral advice [73]. Future maternal health policies should guarantee free timely referral and these could make use of pro-poor health insurance schemes, user-fee exemptions and vouchers [74, 75].

Limited decision-making capacity of women, fear of blood transfusion and fear to die in higher level facilities have been noted by similar studies in sSA where men, in-laws and elders decide whether women are allowed to travel [76–78]. In rural Uganda, women enduring maternity pain were considered brave since pregnancy was perceived as a test of endurance [79]. These women considered referral facilities as the last resort due to preferences for traditional birthing at home [79]. Active community engagement is essential in overcoming these socio-cultural barriers [80]. Evidence indicates that involving men or women’s partners is beneficial because men can escort women if they understand why women are referred [81–83].

Poor performance may be a source of dissatisfaction, especially where shortages of skilled health professionals exist [47–49]. Lack of medical supplies and essential utilities such as electricity and clean water could also result in poor care experiences as reported in South Sudan [84]. Training adequate numbers of maternity care providers and ensuring an equitable distribution can mitigate the challenges confronting quality maternal and newborn referrals [85, 86].

No study in our review investigated women’s experiences in relation with human-centred and dignified care. Women who feel to be respected, treated with equity and offered emotional support have better post-partum psychological outcomes [87]. WHO notes that about 10% of pregnant women and 13% of women just after birth encounter mental health issues globally [88]. These are even higher (15.6% during pregnancy and 19.8% after birth) in sSA LLMICs. This prompts the need for maternity care providers to prioritise emotional wellbeing of women, especially after referral.

Quality referral was interpreted differently in the different studies. Some focused on the ability of healthcare providers to identify and initiate referral, having means of transport and detailed documentation of referrals. These fit well within our conceptual framework as indispensable elements in achieving high-quality maternity referrals [23]. Our findings imply the absence of a standard definition of quality referral, yet these key elements must characterise a maternity referral system that seeks to achieve desirable outcomes and positive maternal experiences. Monitoring and evaluation aimed at determining whether referrals reflect those features are needed to enhance referral [11, 67].

### Strengths and limitations

Our study followed the PRISMA Statements for conducting systematic reviews. The study was underpinned by a framework that provides a balance between embracing quality referrals from the perspectives of both healthcare providers and women. The study was limited, however, to articles published in English in sSA LLMICs.

## Conclusion

Referral guidelines are not always properly implemented due to human resource constraints, referral costs borne by women and their families, poor coordination between levels of care and limited availability of equipment, medicines and transport issues. Governments, planning agencies and healthcare administrators need to focus on more operationally-oriented guidelines to enable health facilities to facilitate maternal and newborn referral. There is a need for well-coordinated and strengthened teamwork and collaboration between PHC, BEmOC and CEmOC providers. Community education and interventions can encourage men to be involved in maternity care. Low cost transport is needed to mitigate barriers for referral. To ensure quality maternal and newborn referral, mechanisms should be instituted for health managers at district level to monitor and evaluate referral documentation, its quality and efficiency regularly.

## Supplementary information

**Additional file 1 S 1.** Framework for assessing the quality of maternal referrals. Source: Adapted from Hulton, Matthews & Stones, 2000.

**Additional file 2 Table 2.** Summary of findings

## Data Availability

All analysed data are included in this article.

## Abbreviations

BEmONC: Basic emergency obstetric and newborn care
CEmONC: Comprehensive emergency obstetric and newborn care
EmOC: Emergency and obstetric care
EmONC: Emergency obstetric and newborn care
FBO: Faith-based organisation
GIS: Geographic information systems
NGOs: Non-governmental organisations
PRISMA: Preferred reporting items for systematic reviews and meta-analyses
sSA-LLMICs: Sub-Saharan African low and lower middle income countries
TBA: Traditional birth attendant
WHO: World health organisation
