# Supplementary material for: Quality and women’s satisfaction with maternal referral practices in sub-Saharan African low and lower-middle income countries: a systematic review
Source: BMC Pregnancy Childbirth. 2020 Nov 11;20:682. doi: 10.1186/s12884-020-03339-3 (PMC7656726; doi:10.1186/s12884-020-03339-3)
Supplement: Supplementary file 1 — Additional file 1 S 1. Framework for assessing the quality of maternal referrals. Source: Adapted from Hulton, Matthews & Stones, 2000. [file 12884_2020_3339_MOESM1_ESM.docx]

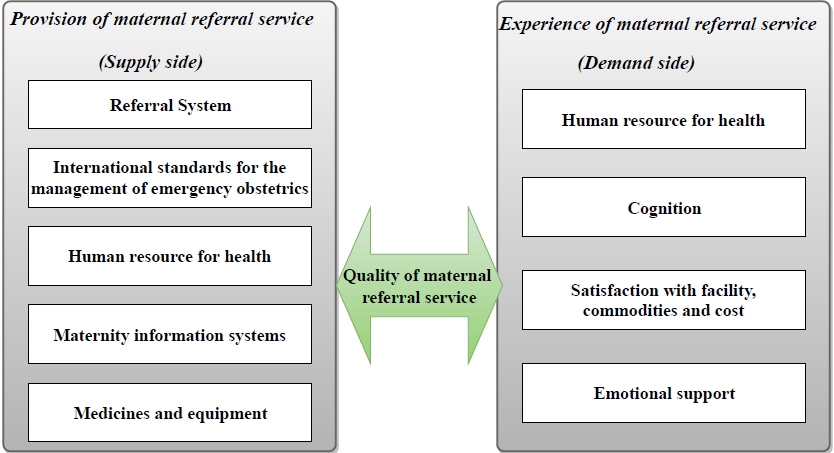


**S 1. Framework for assessing the quality of maternal referrals**

Source: Adapted from Hulton, Matthews & Stones, 2000
