# Supplementary material for: Quality and women’s satisfaction with maternal referral practices in sub-Saharan African low and lower-middle income countries: a systematic review
Source: BMC Pregnancy Childbirth. 2020 Nov 11;20:682. doi: 10.1186/s12884-020-03339-3 (PMC7656726; doi:10.1186/s12884-020-03339-3)
Supplement: Supplementary file 2 — Additional file 2 Table 2. Summary of findings [file 12884_2020_3339_MOESM2_ESM.docx]

**Table 2. Summary of findings**

|  | **Provision of maternal referral service (Supply)** | | | | | **Experience of maternal referral service (Demand)** | | | | |
| --- | --- | --- | --- | --- | --- | --- | --- | --- | --- | --- |
|  | **Referral system** | **International standards for EmOC** | **Human resource for health** | **Maternity information systems** | **Medicines and equipment** | **Human resource for health** | **Cognition** | **Respect, dignity and equity** | **Satisfaction with facility, commodities and cost** | **Emotional support** |
| Abodunrin et al, 2010 |  |  | ✓ |  |  |  |  |  |  |  |
| Afari et al, 2014 | ✓ | ✓ | ✓ | ✓ |  |  |  |  |  |  |
| Akaba & Ekele, 2018 | ✓ |  |  |  |  |  |  |  |  |  |
| Awoonor-Williams et al, 2015 | ✓ | ✓ | ✓ | ✓ | ✓ |  |  |  |  |  |
| Carnahan et al, 2016 | ✓ |  | ✓ |  | ✓ |  |  |  |  |  |
| Elmusharaf et al, 2017 | ✓ |  | ✓ |  |  |  |  |  |  |  |
| Goodman et al, 2017 | ✓ |  |  | ✓ |  |  |  |  |  |  |
| Kyei-Onanjiri et al, 2018 | ✓ | ✓ | ✓ | ✓ |  |  |  |  |  |  |
| Mirkuzie et al, 2016 |  |  | ✓ |  |  |  |  |  |  |  |
| Mselle & Kohi, 2016 |  |  |  |  |  | ✓ |  |  |  |  |
| Nuamah et al, 2016 | ✓ |  | ✓ |  |  |  |  |  |  |  |
| Nwameme et al, 2014 | ✓ | ✓ | ✓ | ✓ |  | ✓ |  |  | ✓ |  |
| Okafor et al, 2015 |  |  | ✓ |  |  |  |  |  |  |  |
| Shimoda et al, 2015 |  | ✓ | ✓ |  | ✓ |  |  |  |  |  |
| Strand et al, 2009 | ✓ |  |  |  | ✓ |  |  |  |  |  |
| Tayler‐Smith et al, 2013 | ✓ |  |  |  |  |  |  |  |  |  |
| Windsma et al, 2017 | ✓ |  | ✓ |  |  |  |  |  |  |  |
